# Self-harm and suicidal behaviour among incarcerated adults in the Australian Capital Territory

**DOI:** 10.1186/s40352-018-0071-8

**Published:** 2018-08-14

**Authors:** Amanda Butler, Jesse T. Young, Stuart A. Kinner, Rohan Borschmann

**Affiliations:** 1 Faculty of Health Sciences, Simon Fraser University, Vancouver, BC Canada; 2 Melbourne School of Population and Global Health, The University of Melbourne, Melbourne, Australia; 3 School of Population and Global Health, The University of Western Australia, Perth, Australia; 4 National Drug Research Institute, Curtin University, Perth, Australia; 5 Centre for Adolescent Health, Murdoch Children’s Research Institute, Melbourne, Australia; 6 Griffith Criminology Institute, Griffith University, Mt Gravatt, Australia; 7 Mater Research Institute-UQ, The University of Queensland, Brisbane, Australia; 8 School of Public Health and Preventive Medicine, Monash University, Melbourne, Australia; 9 Health Service and Population Research Department; Institute of Psychiatry, Psychology & Neuroscience, King’s College London, London, UK; 10 Department of Psychiatry, The University of Melbourne, Melbourne, Australia

## Abstract

**Background:**

Suicide is the leading cause of death in prisons worldwide. Improved understanding of the factors associated with suicide is necessary to inform targeted suicide prevention and interventions. Here we aim to (a) document the prevalence of suicide attempts, suicidal ideation, self-harm, and mental disorder; and (b) identify demographic, mental health, and criminal justice correlates of suicidal ideation, in a sample of incarcerated adults in Australia.

**Methods:**

Data were obtained from the 2016 Detainee Health and Wellbeing Survey conducted in the Alexander Maconochie Centre, the Australian Capital Territory’s only adult prison. Interviews with 98 incarcerated adults were conducted in October 2016. Descriptive statistics were calculated for all measures. Crude differences between participants who reported experiencing suicidal ideation in their lifetime and those who did not were compared using Fisher’s exact test.

**Results:**

Nearly half of the participants (48%, *n* = 47) reported lifetime suicidal ideation and 31% (*n* = 30) reported attempting suicide at some point in their lives. Eighteen participants (18%) reported a lifetime history of having engaged in self-harm. Factors significantly associated with suicidal ideation included lifetime history of mental disorder, self-harm, experiencing a drug overdose, and being hospitalized in the past 12 months.

**Conclusion:**

The burden of suicidal ideation and prior suicide attempts among people in prison is substantial. Incarceration is a pivotal opportunity to identify people with a history of mental health problems and target interventions aimed at reducing adverse outcomes including suicide mortality.

## Background

Suicide is the leading cause of death among incarcerated adults worldwide and preventing suicide in prison has become an international priority (Marzano et al. 2016; World Health Organization 2007). In England and Wales, rates of suicide are five and 20 times higher among incarcerated males and females, respectively, than in the age- and sex-standardized general population (Fazel and Benning 2009; Fazel, Benning and Danesh 2005). In Australia, one study found that the rate of suicide is five and 12 times higher among incarcerated men and women, respectively, compared with the general population (Kariminia et al. 2007). While several countries have developed targeted prevention strategies, the epidemiology of suicide in prison suggests that current strategies are inadequate and must be improved to meet the complex needs of incarcerated adults (Daigle et al. 2007; Marzano et al. 2016).

Suicidal ideation, suicide attempts, self-harm history, and mental health disorders are predictors of future suicide and, as such, are targets for prevention and treatment (Borschmann et al. 2016; Fazel et al. 2008; Skegg 2005). A meta-analysis of nearly 5000 suicide deaths in prisons (mostly in the US) found that the risk of suicide was 15 times higher among those who experienced recent suicidal ideation, and approximately 50% of people who died by suicide in prison had a history of self-harm (Fazel et al. 2008). In an Australian study, 15–21% and 34–44% of people in prison reported a lifetime history of attempted suicide and suicidal ideation, respectively (Larney et al. 2012).

In a study of 26,510 people in prison in England and Wales, Hawton et al. (2014) examined 139,195 self-harm incidents and estimated that 5–6% of males and 20–24% of females in custody self-harmed each year. Furthermore, self-harm was found to be an important risk factor for subsequent suicide deaths during incarceration, with rates of 450 compared to 98 deaths per 100,000 for people with and without a history of self-harm, respectively (Hawton et al. 2014). Self-harm is often repetitive; a meta-analysis of 90 studies from Europe and the UK estimated that at least 15% of people who were hospitalized for self-harm in the community had more than one self-harm event within 1 year of release from hospital, and two-thirds of suicide deaths were preceded by self-harm (Owens et al. 2002). In prison, self-harm increases the likelihood of suicide by 6–11 times compared to people without a history of self-harm (Fazel et al. 2008). Mental disorders are overrepresented among incarcerated adults and mental disorders are strong predictors of both suicide and self-harm (Borschmann et al. 2016; Fazel and Seewald 2012; Prins 2014; Skegg 2005).

Although social exclusion, disadvantage and trauma are common pre-prison adversities, there are also factors associated with the prison environment that have the potential to precipitate or exacerbate mental illness and increase suicide risk. These factors – sometimes referred to as “pains of imprisonment” – may include prison violence, isolation, employment insecurity, lack of privacy, and forced solitary conferment (Armour 2012; Metzner and Fellner 2010).

The aims of the current paper were to (a) document the prevalence of self-harm, suicidal behavior, and mental disorder; and (b) identify demographic, mental health and criminal justice correlates of suicidal behavior, in a sample of incarcerated adults in Australia.

## Methods

Data were obtained from the *2016 Detainee Health and Wellbeing Survey* conducted in the Alexander Maconochie Centre, the Australian Capital Territory’s only adult prison. Detained adults (including those who had been sentenced and those on remand) completed a confidential interview with researchers during October 2016. The survey was administered face-to-face by trained interviewers using secure electronic tablets. The interview covered a range of topics including demographics, social determinants of health, mental health and wellbeing, substance use, health-promoting and health-compromising behaviours, and use of health services inside and outside of prison. Additionally, participants were asked whether they had ever/recently thought about attempting suicide, made one or more suicide attempts, or engaged in self-harm. Participants who reported that they had a history of suicidal ideation, suicide attempts, or self-harm were then asked about the number of previous suicide attempts or self-harm events.

Crude differences between participants who reported experiencing suicidal ideation in their lifetime and those who did not were compared using Fisher’s exact test. Suicidal ideation was established using responses to the survey question: “Have you ever thought about attempting suicide?” Analyses were conducted using SPSS Statistics version 25.0 (IBM Corp. 2017).

## Results

Ninety-eight individuals (24% of the adult prison population in ACT at the time of recruitment) consented and were recruited to the study. Most participants were male (84%) and aged 25 to 44 years (68%); one-third (33%) identified as Aboriginal and/or Torres Strait Islander.

**Fig. 1:**
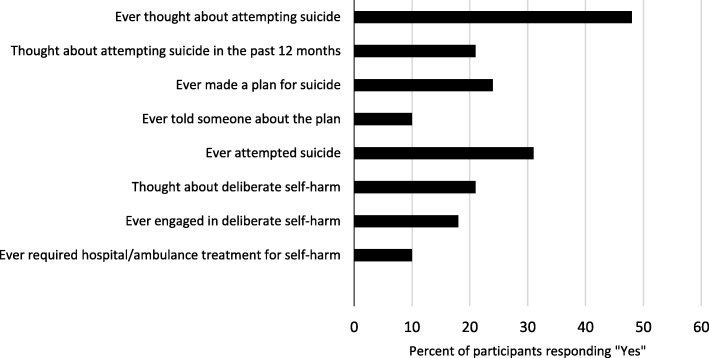
Self-harm and suicidal behaviour among survey respondents (*N* = 98)

Figure 1 presents the proportion of participants who reported prior suicide attempts, suicidal ideation and/or engaging in self-harm. Forty-seven respondents (48%) reported lifetime thoughts of attempting suicide, of whom 24 (51%) reported ‘making a plan’ to end their life; 21 (45%) thought about attempting suicide in the preceding 12 months; and 30 (64%) reported attempting suicide at some point in their lives. Eighteen participants (18%) reported ever having engaged in self-harm, of whom 10 (56%) reported that they required ambulance or hospital contact for a self-harm event in their lifetime. Among those who reported self-harm, the median number of lifetime acts reported was six (range: 1–100).

Fifty-four percent of participants (*n* = 53) reported that they had received at least one mental disorder diagnosis from a GP or mental health professional in their lifetime and 19% (*n* = 19) reported lifetime admission to a psychiatric ward. The most commonly reported mental disorders were depression (*n* = 41; 42%) anxiety (*n* = 28; 29%) substance use disorder (*n* = 26; 27%) and personality disorder (*n* = 16; 16%). Among those who reported a lifetime history of substance use disorder, 62% reported a current substance use disorder.

Table 1 presents factors associated with suicidal ideation. Rates of suicidal ideation did not differ significantly based on gender or Indigenous status. Factors significantly associated with suicidal ideation included a lifetime history of mental disorder, a lifetime history of self-harm, experiencing a drug overdose, being hospitalized (general or psychiatric) in the past 12 months, and lifetime history of being hospitalized in a psychiatric ward. Depression, anxiety, substance use disorder, personality disorder, and schizophrenia were significantly related to suicidal ideation, while bipolar disorder was not. Forty-one percent of people with a lifetime history of suicidal ideation also reported a history of self-harm. Sixty-six percent of those who experienced suicidal ideation also reported attempting suicide at some point in their lives.

**Table 1:** Factors associated with suicidal ideation (*N* = 98)

	Suicidal ideation (N)	Suicidal ideation (Y)	Total sample	*p*-value^a^
	N(%) 42 (42.9%)	N(%) 47(48%)	N(%) *N* = 98	
Gender				
Female	8 (19%)	3 (6.4%)	11 (11.2%)	0.106
Male	34 (81%)	43 (91.5%)	77 (78.6)	
			88 (89.7%)	
Aboriginal status				
Yes	10 (23.8%)	17 (36.2%)	27 (27.6%)	0.252
No	32 (76.2%)	30 (63.8%)	62 (63.2%)	
			89 (90.8%)	
Mental illness (lifetime)				
Yes	16 (38.1%)	35 (74.5%)	51 (52.0%)	0.001
No	26 (61.9%)	12 (25.5%)	38 (38.8%)	
			89 (90.8%)	
Depression (lifetime)				
Yes	9 (21.4%)	30 (63.8%)	39 (39.8%)	<.001
No	33 (78.6%)	17 (36.2%)	50 (51.0%)	
			89 (90.8%)	
Anxiety (lifetime)				
Yes	7 (16.7%)	21 (44.7%)	28 (28.6%)	0.006
No	35 (83.3%)	26 (55.3%)	61 (62.2%)	
			89 (90.8%)	
Bipolar (lifetime)				
Yes	4 (9.5%)	10 (21.3%)	14 (14.3%)	0.154
No	38 (90.5%)	37 (78.7%)	75 (76.5%)	
			89 (90.8%)	
Personality disorder (lifetime)				
Yes	3 (7.1%)	13 (27.7%)	16 (16.3%)	0.014
No	39 (92.9%)	34 (72.3%)	73 (74.5%)	
			89 (90.8%)	
Schizophrenia (lifetime)				
Yes	4 (9.5%)	13 (27.7%)	17 (17.3%)	0.034
No	38 (90.5%)	34 (72.3%)	72 (73.5%)	
			89 (90.8%)	
Substance use disorder (lifetime)				
Yes	6 (14.3%)	19 (40.4%)	25 (25.5%)	0.009
No	36 (85.7%)	28 (59.6%)	64 (65.3%)	
			89 (90.8%)	
Hospitalization in last 12 months (general or psychiatric)				
Yes	5 (12.2%)	16 (34.8%)	21 (21.4%)	0.023
No	36 (87.8%)	30 (65.2%)	66 (67.3%)	
			87 (88.8%)	
Psychiatric hospitalization (lifetime)				
Yes	4 (9.5%)	13 (28.3%)	17 (17.3%)	0.032
No	38 (90.5%)	33 (71.7%)	71 (72.4%)	
			88 (89.8%)	
Heroin use in last 12 months				
Yes	11 (26.2%)	21 (44.7%)	32 (32.7%)	0.081
No	31 (73.8%)	26 (55.3%)	57 (58.2%)	
			89 (90.8%)	
Cocaine use in last 12 months				
Yes	3 (7.1%)	8 (17.0%)	11 (11.2%)	0.205
No	39 (92.9%)	39 (83.0%)	78 (79.6%)	
			89 (90.8%)	
Alcohol use in last 12 months				
Yes	23 (54.8%)	33 (70.2%)	56 (57.1%)	0.187
No	19 (45.2%)	14 (29.8%)	33 (33.7%)	
			89 (90.8%)	
Injected drug use in last 12 months				
Yes	6 (14.3%)	13 (27.7%)	19 (19.4%)	0.195
No	36 (85.7%)	34 (72.3%)	70 (71.4%)	
			89 (90.8%)	
Drug overdose (lifetime)				
Yes	6 (14.6%)	21 (45.7%)	27 (27.6%)	0.002
No	35 (85.4%)	25 (54.3%)	60 (61.2%)	
			87 (88.8%)	
Self-harm (lifetime)				
Yes	0 (0%)	18 (40.9%)	18 (18.4%)	<.001
No	33 (100%)	26 (59.1%)	59 (60.2%)	
			77 (78.6%)	

^a^Fisher’s exact test

Total sums to less than 100% due to missing data

## Discussion

The lifetime prevalence of self-reported suicide attempts and 12-month prevalence of suicidal ideation in this sample of incarcerated adults (30 and 21% respectively) was almost 10 times higher than the estimated prevalence in the general Australian population (3.6 and 2.3% respectively) (Australian Bureau of Statistics 2007; Pirkis et al. 2000).

The lifetime prevalence of self-harm (*n* = 18; 18%) in the sample was consistent with rates found in the general adult population – an Australian community survey found that the lifetime prevalence of self-harm was 24 and 18% among females and males aged 20–24, respectively (Martin et al. 2010). All participants in our study who engaged in self-harm also reported suicidal ideation at some point in their lives.

Upon reception into prison, people with a history of self-harm should be considered at elevated risk of suicide, with particular attention paid to those with a history of repeated self-harm (Hawton et al. 2014). Given that most people who self-harm do not seek help (Rowe et al. 2014), incarceration is a unique opportunity to identify self-harm history and initiate appropriate mental health treatment. Self-reported self-harm lacks sensitivity, however identification of people at risk of self-harm can be improved through combining multiple data sources, including community health records (Borschmann et al. 2017).

It has been estimated in Australia that the risk of self-harm *after* release from prison is three times greater than for those who are still incarcerated (Borschmann et al. 2016), making the transition from prison into the community a critical period for the prevention of mortality and morbidity.

Over half of the participants in our study reported being diagnosed with a mental disorder at some point in their lifetime. Depression was the most frequently reported disorder and was significantly associated with suicidal ideation. Depression has been identified as an important risk factor for suicidal behaviour and is the most common psychiatric disorder diagnosed in people who die by suicide (Hawton et al. 2013). As such, obtaining a comprehensive mental health history at prison reception is important for assessing suicide risk. Among participants who reported a mental disorder diagnosis in this study, half also reported a comorbid substance use disorder. Substance use disorder was associated with increased suicidal ideation which is consistent with the literature (Fazel et al. 2008). These findings underscore the critical importance of providing integrated mental health and substance use treatment for people with dual diagnoses who experience incarceration. Bipolar disorder was not found to be related to suicidal ideation in univariate analyses. Fazel et al. (2013) found that the risk of suicide was higher for people in prison with bipolar disorder compared to people with no psychiatric disorder, but the risk was much lower than for other psychiatric disorders including depression and schizophrenia-spectrum disorders. Further research is required to confirm associations and potential modifiers of these associations.

The World Health Organization (2007) recommends that screening for risk should take place at intake and at regular intervals during incarceration and suicide prevention should involve ongoing observation by trained health and custodial staff. One study from the United States found that juvenile detention facilities that used screening for everyone within 24 h of admission to custody had a 55% reduction in suicide attempts compared to facilities that did not screen (Gallagher and Dobrin 2005).

This is the first ever study of self-harm and suicidal ideation among people incarcerated in the Australian Capital Territory. The study had a few notable limitations. First, we used convenience sampling, thus the prevalence reported here cannot be considered representative of the prevalence in the wider Australian prison population. Second, as self-harm is typically under-reported in the prison environment (Borschmann et al. 2017), it is likely that we under-estimated the lifetime prevalence of self-harm. Disclosure of self-harm can be highly stigmatising (Mackay and Barrowclough 2005) and may result in increased correctional monitoring and restrictive interventions (Justice Health 2015). Third, while we did not find significant differences in suicidal ideation related to drug use in the past 12 months, this may be impacted by restricted access to illicit drugs while in custody. Finally, although we recruited 24% of the incarcerated population in the ACT, our modest sample size precluded multivariate or sex-stratified analyses.

## Conclusion

Our study adds support to previous studies which have reported that the burden of suicidal ideation and prior suicide attempts among people in prison is substantial (Fazel and Benning 2009; Fazel et al. 2005; Fazel et al. 2008; Hawton et al. 2014). To minimise the risk of suicidal behaviour after release from prison, people at risk should be systematically identified, and individually-tailored, continuous care pathways between prison- and community-based health-care services should be clearly defined, complemented by meaningful investment in evidence-based transitional support (Borschmann et al. 2018). Interventions targeted at modifiable risk factors for suicide and self-harm have the potential to reduce adverse outcomes including suicide mortality. These efforts must be informed by a better understanding of the factors that put people at higher risk.
